# Reflections on modern methods: linkage error bias

**DOI:** 10.1093/ije/dyz203

**Published:** 2019-10-21

**Authors:** James C Doidge, Katie L Harron

**Affiliations:** 1 Intensive Care National Audit and Research Centre, London, UK; 2 UCL Great Ormond Street Institute of Child Health, University College London, London, UK

**Keywords:** Linkage error, record linkage, data linkage, bias, information bias, selection bias, sensitivity analysis, bias analysis, quantitative bias analysis, missing data

## Abstract

Linked data are increasingly being used for epidemiological research, to enhance primary research, and in planning, monitoring and evaluating public policy and services. Linkage error (missed links between records that relate to the same person or false links between unrelated records) can manifest in many ways: as missing data, measurement error and misclassification, unrepresentative sampling, or as a special combination of these that is specific to analysis of linked data: the merging and splitting of people that can occur when two hospital admission records are counted as one person admitted twice if linked and two people admitted once if not. Through these mechanisms, linkage error can ultimately lead to information bias and selection bias; so identifying relevant mechanisms is key in quantitative bias analysis. In this article we introduce five key concepts and a study classification system for identifying which mechanisms are relevant to any given analysis. We provide examples and discuss options for estimating parameters for bias analysis. This conceptual framework provides the ‘links’ between linkage error, information bias and selection bias, and lays the groundwork for quantitative bias analysis for linkage error.

**Key Messages**

- Linkage error can manifest as missing data, misclassification or measurement error, or erroneous inclusion or exclusion of people from an analysis. It can also cause splitting of one person’s records into multiple units of observation, and merging of multiple units into one.
- Misclassification and measurement error can lead to information bias. Rates of misclassification and measurement error may be higher when links are meaningfully interpreted, such as when deriving vital status from linkage to a register of deaths.
- When inclusion or exclusion from an analysis rely on accurate linkage, linkage errors may lead to selection bias.
- When units of observation cannot be uniquely identified without linkage (e.g. analysis of people within a set of event records), linkage errors may lead to splitting and merging. Splitting and merging may lead to both information bias and selection bias.
- Considering only the links between two sets of records and the sampling frame for an analysis, there are 11 possible linkage structures that can help to identify the qualitative manifestations of linkage error, but linkage errors within each set may also need to be considered.

## Introduction

**Figure 1.**
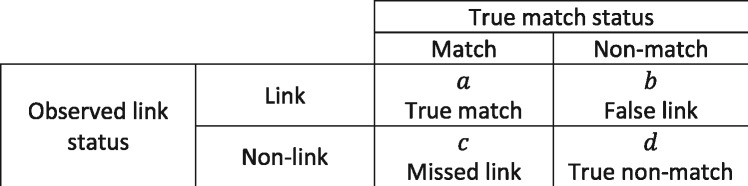
2 × 2 table representing accuracy in record linkage. As with screening tests, linkage accuracy can be represented in a 2 × 2 table where sensitivity (or recall) = *a*/(*a* + *c*) and specificity = *b*/(*b* + *d*), positive predictive value (or precision) = *a*/(*a* + *b*) and the negative predictive value = *d*/(*c* + *d*).

With advances in computing technology and increasing use of secondary data for research, there has been rapid growth in analysis of linked data but little corresponding acknowledgement of the statistical problems introduced by linkage error: missed links between records relating to the same entity (usually a person) and false links between records relating to different entities (Figure 1). This article explores the different ways in which linkage error can manifest in linked data (as missing data, measurement error and misclassification, and as distortions in the representativeness of a sample) and how these different manifestations ultimately lead to information bias and selection bias. We aim to help users of linked data implement quantitative bias analysis^1^^,^^2^ for linkage error, but start by introducing three key concepts and a study classification system that help to identify the qualitative manifestations of linkage error. We proceed to introduce two more concepts that define quantitative aspects of linkage error that may be required for bias analysis, and summarize the techniques available for estimating these. We discuss published examples of linked data analysis to illustrate key concepts and different manifestations of linkage error. For introductions to methods for implementing record linkage, see Doidge and Harron,^3^ Harron^4^ or Winkler.^5^ New terms and those with specific meanings are summarised in the glossary.

## Linkage error within a framework for bias analysis

Rothman and colleagues^6^ identify three fundamental mechanisms through which bias can arise: information bias, selection bias and confounding. Their framework focuses on bivariate statistics (effect measures etc.) and the concept of confounding is specific to these. Information bias and selection bias, however, reflect limitations of data quality in terms of accuracy and representativeness and are relevant to both univariate statistics (prevalence etc.) and bivariate statistics. This section explores the different ways that linkage error can manifest in a dataset, highlighting how each is relevant to these concepts of information bias and selection bias.

Information bias arises from measurement error in quantitative variables or misclassification in categorical variables. One of the more straightforward impacts of linkage error is when a false link results in incorrect information being obtained from a record that belongs to a different entity. For example, if we link together reading and mathematics scores for two different children, we would introduce measurement error unless the two children happened to have the same scores. There are many situations in which missed links can also result in incorrect information being derived; this is especially likely when only a subset of records are expected to have links, and the presence or absence of a link is meaningfully interpreted, such as when we infer mortality from linkage to a register of deaths. In this case, it is not the data contained in the death record per se that provides information, but the existence of the link itself.

When linkage is meaningfully interpreted, missed links and false links can both lead to misclassification. The misclassification, however, operates in opposite directions, so missed links and false links can offset each other’s influence. For example, missed links to a register of deaths would cause false-negative misclassification of mortality, whereas false links could cause false-positive misclassification.

When linkage is not meaningfully interpreted, missed links result in missing data and false links result in potential misclassification or measurement error. Note the caveat here; false links only lead to misclassification or measurement error when the information contained in the falsely linked records differs from the information that would have been derived from correctly linked records. For example, a link from one dead person to another person’s death record would not result in misclassification of vital status, but it might result in measurement error in time to death. This potentially important caveat requires many of the statements in this section to be caged in uncertain terms (can, could etc.) and its relevance to bias analysis will be discussed in the next section.

**Key concept 1**: Links can be meaningfully interpreted to imply the value of some variable. When links are meaningfully interpreted, both missed links and false links can manifest as misclassification or measurement error in that variable, but in opposite directions.

Selection bias occurs when the probability of inclusion in an analysis is correlated with one or more of the variables of interest.^6^ There are three ways that linkage error can influence inclusion in an analysis. First, a characteristic that is obtained through linkage may itself be a criterion for inclusion or exclusion, such as inclusion of people with links to a disease register or exclusion of those with links to a register of deaths. In these cases, linkage is meaningfully interpreted with respect to the inclusion criteria (e.g. having a particular disease or being alive), but not with respect to any variable of interest. Thus, whereas there is misclassification occurring, it is introducing error into the sampling frame of the analysis, rather than into the variables of interest. It therefore operates functionally as a form of selection bias rather than information bias, and this affects how the bias should be corrected.^1^

When linkage is not meaningfully interpreted and missed links lead to missing data, then how those missing data are handled determines the implications of linkage error. Invalid techniques for imputing missing data can induce information bias, and another common strategy for addressing missing data is exclusion (listwise deletion or complete case analysis). Exclusion of individuals with data that are missing introduces potential for selection bias when missing data from missed links are not missing completely at random (i.e. when the probability of missed links depends on one or more variable of interest).^7^

The third way that linkage error can affect inclusion in an analysis is more abstract; the double-counting that can occur when missed links cause one entity’s records to be split into multiple apparent entities, and the undercounting that can arise when records relating to separate people are inappropriately merged because of a false link. Double-counting and undercounting can be operationalized as representing relative selection probabilities of greater than one or less than one, respectively.

**Key concept 2**: When selection depends on the accuracy of linkage, linkage error may lead to selection bias. This can happen because linkage error leads: to misclassification or measurement error in selection criteria; to missing data in records that are subsequently excluded; or to splitting and merging.

This splitting and merging of entities often involves some degree of both information bias and selection bias. For example, depending on whether they are linked, two hospital admission records may be counted as either one person admitted twice or as two people admitted once. Misclassification or measurement error may be implicated whenever variables of interest are derived from multiple records. In the hospital example, readmission statistics could be affected, but demographic characteristics that were constant across records or were derived from a single record would not be. Analyses involving variables derived from multiple records are therefore particularly susceptible to bias from merging and splitting.

Merging and splitting is a concern whenever the target sample for an analysis is not uniquely identified in the data, prior to linkage. If a sample is to be drawn from a single, event-based file that must be ‘internally linked’ to enable analysis at the person level, then the units of analysis (people) can be affected by linkage error. Even when both files in a linkage contain only a single record for each entity, if the sampling frame includes people from either file then the sample cannot be uniquely identified until after linkage and the potential for merging and splitting remains. A missed link in these situations could result in somebody being counted twice (once in File A only and once in File B only) and a false link could result in two different people being counted once (as one person appearing in both files). The sample can only be uniquely identified prior to linkage when it is drawn from a single file that does not itself require internal linkage.

**Key concept 3**: Unless the sample is uniquely identified prior to linkage, linkage error may lead to splitting and merging of entities (units of observation). Splitting and merging can be operationalized as a combination of varied probabilities of selection and misclassification or measurement error in variables that are meaningfully interpreted or otherwise derived from multiple records.

Establishing the potential for merging and splitting requires careful consideration of the unit of observation. A set of hospital admission records, for example, may contain a uniquely identified sample if the unit of observation is admissions, but not if the unit is people, and not if the unit is sequenced events such as people’s first admission (because first admissions cannot be identified until they have been linked to any other relevant admissions).

These three key concepts provide three questions in identifying the manifestations of linkage error: (i) are links being meaningfully interpreted? (ii) is selection dependent on linkage? (iii) is there a possibility of splitting or merging? The answers to these are not always straightforward, especially in the case of establishing the potential for merging and splitting within an internally linked file, as discussed above. For links between multiple sets of records (usually representing multiple files but potentially multiple subsets of records from within the same file) we have found that it helps illustrate the sampling frame using a Venn diagram with shading in the region from which the analysis set (sample) is selected.

**Figure 2.**
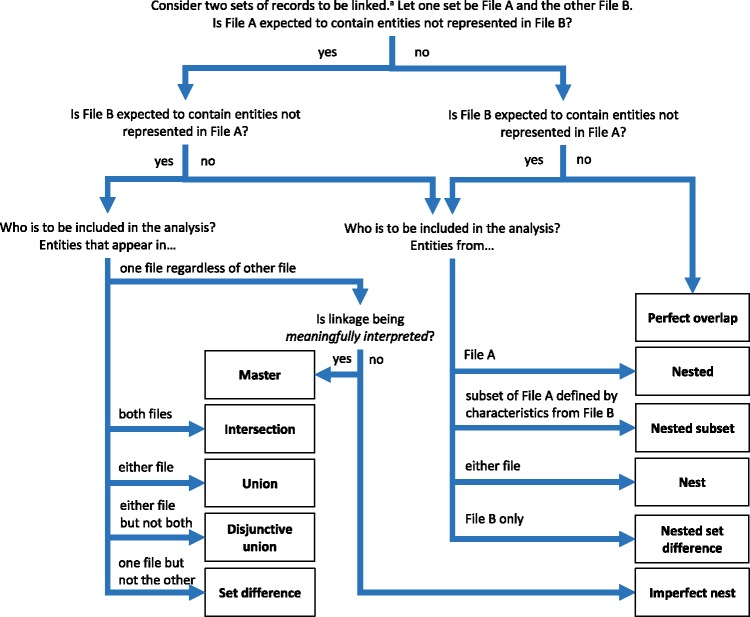
Linkage structure classification tree. ‘Entities’ are the unit at which linkage occurs; usually people but potentially families, households, companies etc. ‘Sets’ refers to groups of records being linked; these may be separate data sources, subsets of larger source files (e.g. hospital admissions for disease X) or even subsets of the same source file (e.g. ‘hospital admissions for disease X’ and ‘possible readmissions’, or linkage of mothers to babies in Hospital Episode Statistics^10^). Linkage within either set can have additional implications for how linkage error can manifest, especially with respect to potential for splitting and merging (see text).

Any two sets can intersect in three possible ways: (i) each set contains the same entities (their coverage overlaps perfectly); (ii) one set contains entities not included in the other (the latter is nested within the former); or (iii) each file contains entities not included in the other (their coverage intersects). Considering the different possible regions within these which could form the sampling frame for an analysis, we have identified 11 possible linkage structures (Table 1; studies that involve more than two files or subsets can be illustrated using combinations of these). For each linkage structure, the answers to the questions above differ and so do the qualitative manifestations of linkage error. Because the linkage structure partly reflects the sampling frame, different analyses of the same linked data may have different linkage structures. A decision tree is provided in Figure 2 to help identify which linkage structure or combination of structures is relevant to a particular analysis. Beware though, that linkage within each set is often also implicated and is not as easy to interpret graphically. The implications of any internal linkage with respect to unique identification of the sample, and the associated risk of merging and splitting, should be considered in addition to the implications listed in Table 1.

**Table 1.** Eleven ‘linkage structures’ for classifying analysis of two linked sets of records

Linkage structure	Venn diagram^a^	Example	Is linkage meaningfully interpreted?	Is splitting or merging possible?	Is selection dependent on linkage?	What are the implications of a missed link?	What are the implications of a false link?
‘Master’	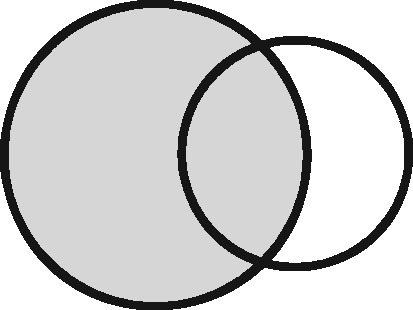	Analysis of mortality risk through linkage to a register of deaths	Yes, with respect to a variable of interest	No	No	False-negative misclassification	Potential false positive misclassification
‘Intersection’	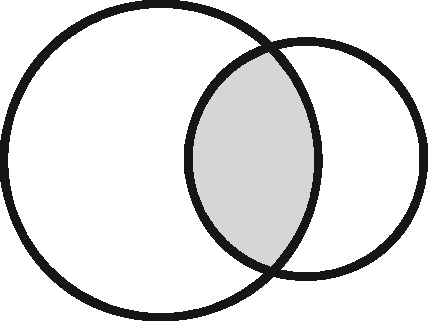	Analysis of health in aeroplane passengers through linkage of health care service data to passenger manifests	Yes, with respect to the inclusion criteria	No	Yes	Erroneous exclusion	Potential erroneous inclusion
‘Union’	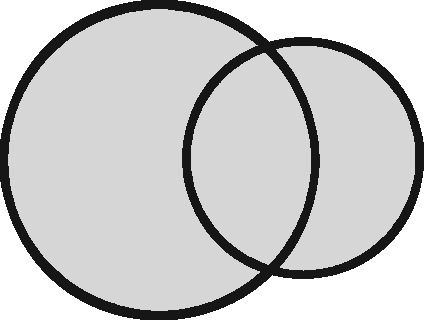	Analysis of pooled data from two providers of a comparable service	Only with respect to variables based on inclusion in both datasets	Yes	Only with respect to potential merging and splitting	Splitting	Merging
‘Disjunctive union’^b^	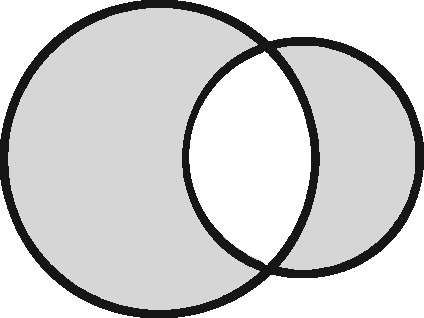	When comparing two services, an analyst may wish to exclude people who used both	Yes, with respect to a variable that is both a criterion for inclusion and a variable of interest	Yes	Yes, with potential for erroneous inclusion of split entities and exclusion of merged entities	Splitting and erroneous inclusion in both subgroups	Merging and erroneous exclusion
‘Set difference’^b^	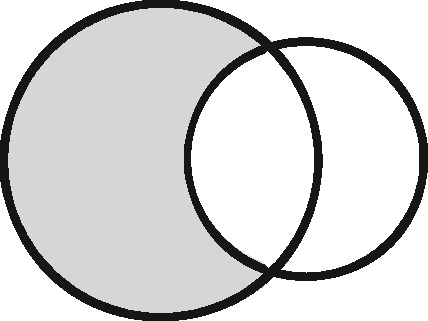	When evaluating one service, an analyst may wish to exclude people who also used an alternative service	Yes, with respect to exclusion criteria	No	Yes	Erroneous inclusion	Potential erroneous exclusion
‘Perfect overlap’^b^	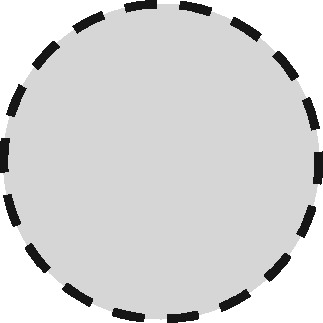	Analysis of data from two services that independently cover the same population, such as one for mothers and one for babies (if every baby record has a corresponding maternal record)	No	No	Only with ‘complete case’ approaches to missing data	Missing data	Potential misclassification or measurement error
‘Nested’	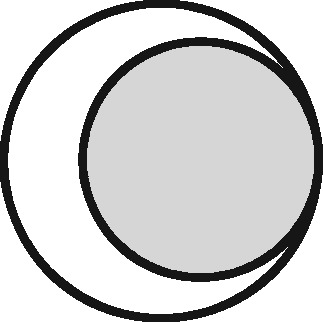	Analysis of birthweight for participants in a cohort study through linkage with birth registrations	No	No	Only with ‘complete case’ approaches to missing data	Missing data	Potential misclassification or measurement error
‘Nested subset’^b^	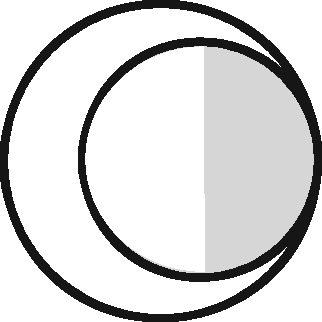	A special case of the nested structure, in which the larger auxiliary file provides information about inclusion or exclusion criteria, e.g. linkage to of a cohort to a birth register, to define a substudy of cohort members with low birthweight	No	No	Yes	Missing data in the selection criteria (which may mean exclusion)	Potential erroneous inclusion or exclusion
‘Nest’	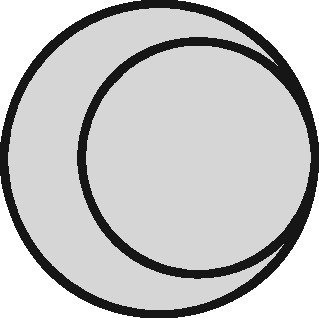	Comparison of outcomes between admitted patients with and without linked test results	Yes, with respect to a variable of interest	No	No	False-negative misclassification	Potential-false positive misclassification
‘Nested set difference’^b^	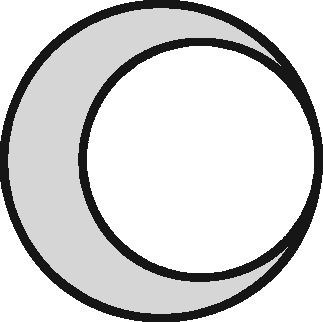	Analysis excluding people who used a service that is only provided to a subset of the population covered by the primary file, e.g. exclusion of patients who received a treatment that was recorded separately	Yes, with respect to criterion for exclusion	No	Yes	Erroneous inclusion	Potential erroneous exclusion
‘Imperfect nest’^b^	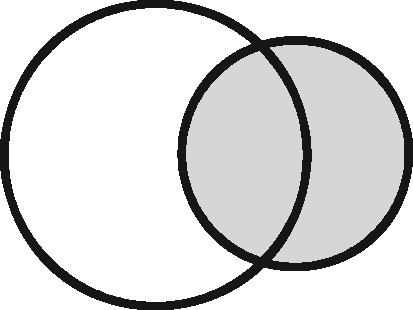	A special case of a nested structure, in which the larger auxiliary file has less than full coverage of the primary file, e.g. linkage to birth records for a cohort that includes some people born overseas.	No	No	Only if a complete case analysis approach is taken to missing data^c^	Missing data	Potential misclassification or measurement error

a Circles represent the population covered by two sets of records, ignoring linkage within either set. Shading represents the region from which the analysis sample is derived (the sampling frame). The size of the circles is irrelevant. Linkage with either set (‘internal linkage’) can have implications that must also be considered (see text).

b In our experience, these structures are unusual in practice; if following the decision tree then revisit questions and ensure responses are appropriate.

c A complete case approach to missing data in an ‘imperfect nest’ structure becomes equivalent to an ‘intersection’ structure.

## Quantitative assessment of linkage error and bias analysis

The previous section explored qualitative differences in the way that linkage error can manifest in different analyses: as misclassification and measurement error, varied probabilities of selection into an analysis, missing data and splitting and merging. In this section we turn to measuring or estimating the quantitative aspects of linkage error which may be needed for bias analysis.

Although the overall rates of missed links and false links are obviously relevant, a key determinant of selection bias and information bias is the distribution of errors with respect to variables of interest.^6^^,^^7^ Selection probabilities that are not associated with variables of interest generally do not induce bias. Similarly, non-differential misclassification (misclassification that is not associated with variables of interest) is generally less of a concern than differential misclassification (although both can cause bias), and data that are missing at random are more amenable to statistical adjustment than data that are missing not at random. It follows that linkage error that is associated with variables of interest induces misclassification, measurement error, missing data, or selection probabilities that are associated with those variables of interest, and generally has greater potential for bias.

**Key concept 4:** Linkage error bias depends on the rates of missed links and false links and the distribution of linkage errors according to variables of interest.

Table 2 provides a list of available techniques for estimating rates of linkage errors or gaining some evidence about the distribution of errors with respect to variables of interest. More information about each technique can be found in the cited literature.

**Table 2.** Techniques for estimating linkage error bias parameters

Technique	False links		Missed links		Limitations
	%	Δ	%	Δ	
Comparison of linked data with training data or ‘gold standard’ (often a subset), e.g. records with unique identifiers available for linkage^14^	✓	✓	✓	✓	Training data that are representative in terms of the quality of matching variables and the association of quality to variables of interest, are rarely available
Negative controls (a subset of records that should definitely not link, i.e. a partial gold standard set), e.g. people known to be alive when linking to a death register^8^ or birth termination records to liveborn babies^15^	✓	✓	✗	✗	Negative controls can be easier to source than positive controls but still require representativeness
Comparison of linked and unlinked records, e.g.^14^	✗	✗	∼^a^	✓	Only useful when expecting ∼100% match rate in one file. No guarantee that linked records are true matches
Comparison of linkable and unlinkable records or records with higher quality matching data and records with lower quality matching data, e.g. missing NHS numbers^16^	✗	✗	✗	✓	Usually feasible, given access to record-level information about matching variable quality
Comparison of plausible and implausible links, e.g. simultaneous admissions to hospital^17^	∼^b^	✓	✗	✗	Often feasible but implausible links are often excluded by data linkers during ‘quality assurance’
Analysis of observed versus plausible number of candidate links, across deterministic rules or probabilistic match weight thresholds, e.g.^18^	✓	✗	✗	✗	Only feasible in 1:1 or 1:many linkages (where at most one link is expected in one or both directions)
Comparison of characteristics of linked data to reference statistics from external data sources, e.g.^19^	∼^c^	∼^c^	∼^c^	∼^c^	Requires representativeness and consideration of other possible reasons for differences, such as differences in data collection and quality

%, can provide evidence about rates of linkage error; Δ, can provide evidence about differences in error rates with respect to variables of interest.

a If 100% of records in one file are expected to link, and the number of false links can be estimated then number of non-links approaches the number of missed links can be derived from these (e.g. if approximately nil false links then the number of missed links is approximately the number of non-links).

b Implausible links usually represent only the ‘tip of the iceberg’ and hide a larger proportion of plausible false links. For some of these, the proportion of all possible scenarios that would be considered implausible can be calculated and used to inversely weight the observed number of implausible links, to estimate the unobserved total number of false links.

c The extent to which this technique can be used to inform estimation of bias parameters depends heavily on representativeness and the absence of any other reasons for observed differences. It is perhaps more useful for qualitative validation than informing quantitative bias analysis, but is sometimes useful.

A recurring caveat in the preceding section was that false links generally only lead to potential measurement error or misclassification, i.e. only when the false link is made to a record containing incongruent values for a variable of interest. All else being equal, false links are more likely to occur to records with frequently occurring values, which is why analyses of rare conditions can be more sensitive to linkage error than analyses of common conditions.^8^

Sometimes, this caveat must be applied to both false links and missed links. When the same information can be derived from multiple records within an entity’s set of matching records, then missing any one of those records may not result in misclassification. For example, if deriving a binary indicator of readmission, then somebody who is readmitted twice would not be misclassified if only one of those readmissions was missed.

Similar caveats are also required for handling linkage errors in the context indirect links (links between records A and B, and records B and C, which create an indirect link between records A and C). A missed link between records A and C may be of no consequence if there is an indirect link formed by links between A and B, and B and C. In essence, there are multiple possible ways for the same information to be derived from records A and C; either through a direct link between these records, or through an indirect link via record B.

These caveats can all be parameterized in the same way, as the distribution of differences between the observed values derived from linked records, and the values that would have been derived from the (truly) matched records.

**Key concept 5:** Linkage errors only have a meaningful impact on data quality when the information derived from the erroneously linked or unlinked records differs from the information that would have been derived from correctly matched records. Linkage error bias therefore depends on the proportion of each type of linkage error that results in incongruent information, and/or the distribution of differences in values between the (observed) linked records and (unobserved) matched records.

Obviously, estimating this distribution of differences is problematic. However, it may often be reasonable or sufficient to assume that all linkage errors lead to meaningful differences (e.g. all missed links to a register of deaths leading to false-negative misclassification of mortality). Furthermore, some of the techniques listed in Table 2 would only detect linkage errors that do lead to meaningful differences, in which case estimating these would suffice. In other cases, estimates of the likely characteristics of erroneously linked records may be obtained by examining the observed characteristics in one of the record sets, and combining this with any available evidence or assumptions about the distribution of errors with respect to these characteristics.

Boxes 1 and 2 provide examples of how linkage error can manifest in different ways in different analyses. Each describes a different linkage structure for combining two data sources, but also highlights the relevance of the caveats described above and of any internal linkage within each data source that may also be required.

**Box 1.** Linkage error in a ‘master’ linkage structure

Scenario: linkage of children admitted to a paediatric intensive care unit (PICU) to a national infection surveillance system, to determine rates of bloodstream infection in PICU patients (see^9^ for complete example).
Linkage structure: ‘master’: the PICU dataset is the master file, which determines the study sample. The datasets do not completely overlap; children in PICU may or may not appear in the infection surveillance file (depending on whether they had an infection or not), and the infection surveillance file includes people who were not admitted to PICU.
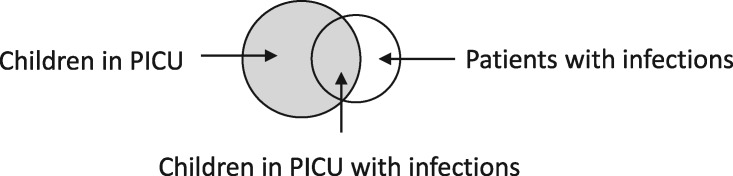
Is the target sample uniquely identified in the data, prior to linkage? Generally, yes; there exists one record per admission in the PICU data and each infection record can link to at most one PICU record. There is no possibility of splitting or merging of admissions. However, if the PICU file were also internally linked, for example to include only one admission per child, then splitting and merging could be implicated. Is linkage meaningfully interpreted? Yes; a link is interpreted as meaning that a child in PICU had an infection, which is a variable of interest. Absence of a link is interpreted as implying that they were infection-free. Is selection dependent on linkage? No; selection into the analysis sample is solely determined by inclusion in a primary file (PICU admissions).
Implications of linkage error: missed links will generally lead to false-negative misclassification of infection status and an underestimation of infection rates (information bias). False links will generally lead to false-positive misclassification and an overestimation of infection rates. Caveats apply to both of these, because of the potential for each admission to have multiple linked records of infection, so consideration should be given to the proportion of missed or false links that are likely to lead to misclassification. Analysis of risk factors for infection could be affected by any differences in rates of linkage error across subgroups or covariates. Information or assumptions about the association of linkage errors with child risk factors and covariates will therefore be critical for analysis.

**Box 2.** Linkage error in an ‘intersection’ linkage structure

Scenario: linkage of electronic flight records with hospital data to evaluate the relationship between length of flight and deep vein thrombosis (see^10^ for complete example).
Linkage structure: ‘intersection’. Hospital data would include people who have not recently flown, and electronic flight records would include people who did not go to hospital. Only linked records are included in the analysis.
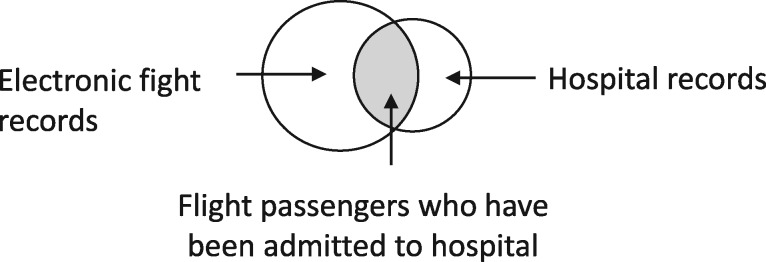
Is the target sample uniquely identified in the data, prior to linkage? Yes. Length of flight is a flight-level characteristic, and the unit of observation must be ‘person-flights’, which would be uniquely identified in the electronic flight records without any possibility of splitting or merging—unless, that is, some further restriction based on internal linkage (e.g. limiting the analysis to one person-flight per person) is also being applied. Is linkage meaningfully interpreted? Yes, with respect to inclusion criteria. Is selection dependent on linkage? Yes, because linkage is meaningfully interpreted with respect to inclusion criteria.
Implications of linkage error: missed links will lead to erroneous exclusion. False links will lead to potential erroneous inclusion (if the flight was taken by somebody who truly did not have a hospital record) and potential misclassification or measurement error in health outcomes (if the health outcomes differed between the falsely linked record and the record that should have been linked). Therefore, both information bias and selection bias could be implicated.

## Discussion

We have identified three key concepts for determining the qualitative manifestations of linkage error, and two that relate to quantitative aspects that require measurement or estimation in bias analysis. Estimating and modelling every potentially relevant bias parameter will often be beyond the realm of feasibility; a balance must be struck between the requirement for accurate estimation, the availability of evidence to inform assumptions, the time required to collect that evidence and incorporate it, and the risk of ambiguity and human error that can be introduced by overly complex analysis.^2^ Simple, best and worst case scenarios are often sufficient to provide bounds of plausibility, provided that the key sources of potential bias can be identified. Many linkage algorithms are designed to maintain precision at a very high level, so false links are often rare enough to justifiably ignore. Engagement between data analysts and data linkers is essential to ensure that assumptions about linkage error are plausible.

Perhaps the biggest limitation of this conceptual framework is that it is rooted in what we (the authors) think of as ‘deterministic analysis’ of linked data; analysis that treats every pair of records as being either linked or not linked.^3^ Just as uncertainty about missing data can be handled using probabilistic techniques such as inverse probability-weighting and multiple imputation, so too can these techniques be applied to analysis of linked data.^11^^,^^12^ There has also been some development of linkage error-adjusted regression estimators.^13^ These are all relatively novel methods, each with different limitations to address and software to develop before they can be widely implemented and validated.

We hope that this framework and classification system help to increase understanding of linkage error, and help researchers address bias in analysis of linked data. The next steps required are the development of generalizable formulae and software tools to make it easier to put these principles into practice.

## Glossary

 *Analysis model*: the statistical model used to estimate parameters of interest; usually does not involve matching variables, like name and address.

*Deterministic linkage*: linkage algorithms based on rules of agreement over matching variables, e.g. ‘agrees on name and date of birth and postcode’.

*Entity*: a distinct unit of observation in an analysis; usually is a person but potentially a family, household, company etc.

*False link*: a link between records that relate to different entities.

*Information bias*: bias induced by misclassification or measurement error.

*Linkage structure*: the relationship of the sampling frame of analysis to the files or subsets being linked, which for any combination of two sets of records can be classified into one or more of the structures described in Table 1 and Figure 2.

*Meaningfully interpreted*: when the presence or absence of a link determines the value for some characteristic, e.g. using linkage with a death register to determine whether someone is dead or alive. This characteristic may be a variable of interest or a criterion for inclusion or exclusion from analysis.

*Measurement error*: error in the value of a quantitative variable.

*Merging*: when two or more entities are counted as one because of at least one false link between their records.

*Misclassification*: error in the value of a categorical variable.

*Missed link*: two records that relate to the same entity but are not linked.

*Missing at random*: the assumption or condition that the probability of data being missing does not depend on the value of the data being analysed, given the data that are observed.

*Missing completely at random*: the assumption or condition that the probability of data being missing does not depend on the value of the data being analysed.

*Missing not at random*: the assumption or condition that the probability of data being missing does depend on the value of the data (alternatively: conditions violating the missing at random or missing completely at random assumptions).

*Non-differential*: does not vary with respect to variables in the analysis model.

*Probabilistic linkage*: linkage algorithms based on scores for patterns of agreement over matching variables, which correlate with the probability that record pairs exhibiting that pattern relate to the same entity.

*Selection bias*: bias induced by differences in the probability of being included in an analysis.

*Selection that depends on linkage*: when linkage and linkage error directly influence the probability of being included in an analysis. See text for explanation of three possible mechanisms through which this can occur.

*Splitting*: when one entity is counted as two or more because of missed links among their records.

*Uniquely identified*: when each entity in the target sample exists as a distinct unit within the data prior to linkage, without any potential for linkage errors to result in merging or splitting.

*Variables of interest*: variables included in the analysis model; not used purely for matching/linkage.
