# Corrigendum to: Reflections on modern methods: linkage error bias

**DOI:** 10.1093/ije/dyaa028

**Published:** 2020-02-24

**Authors:** James C Doidge, Katie L Harron

First published: 21 October 2019, *Int J Epidemiol* 2019;48:2050–60. doi:https://doi.org/10.1093/ije/dyz203.

In the originally published version of this article, there was an error in the Figure 1 caption: “specificity = *b*/(*b*+*d*)” should have read “specificity = *d*/(*b*+*d*)”. Additionally, Box 2 contained a typographic error: “Electronic fight records” should have read “Electronic flight records”. These errors have been corrected.

